# Reducing Pediatric ED Length of Stay by Reducing Diagnostic Testing: A Discrete Event Simulation Model

**DOI:** 10.1097/pq9.0000000000000396

**Published:** 2021-03-10

**Authors:** Kenneth W. McKinley, James M. Chamberlain, Quynh Doan, Deena Berkowitz

**Affiliations:** From the * Emergency Medicine Section of Data Analytics, Children’s National, Washington, D.C.; † Division of Emergency Medicine, Department of Pediatrics, British Columbia Children’s Hospital, Vancouver, BC, Canada

## Abstract

Quality improvement efforts can require significant investment before the system impact of those efforts can be evaluated. We used discrete event simulation (DES) modeling to test the theoretical impact of a proposed initiative to reduce diagnostic testing for low-acuity pediatric emergency department (ED) patients.

**Methods:**

We modified an existing DES model, built at another large, urban, academic pediatric ED, to forecast the impact of reducing diagnostic testing rates on mean ED length of stay (LOS). The modified model included local testing rates for Emergency Severity Index (ESI) 4 and 5 patients and additional processes defined by local experts. Validation was performed by comparing model output predictions of mean LOS and wait times to actual site-specific data. We determined the goal reduction in diagnostic testing rates using the Achievable Benchmark of Care methodology. Model output mean LOS and wait times, with testing set at benchmark rates, were compared to outputs with testing set at current levels.

**Results:**

During validation testing, model output metrics approximated actual clinical data with no statistically significant differences. Compared to model outputs with current testing rates, the mean LOS with testing set at an achievable benchmark was significantly shorter for ESI 4 (difference 19.1 mins [95% confidence interval 12.2, 26.0]) patients.

**Conclusion:**

A DES model predicted a statistically significant decrease in mean LOS for ESI 4 pediatric ED patients if diagnostic testing is performed at an achievable benchmark rate compared to current rates. DES shows promise as a tool to evaluate the impact of a QI initiative before implementation.

## Abstract 2

Supplemental Digital Content is available in the text.

## INTRODUCTION

### Background

Physicians exhibit considerable variation in diagnostic testing for low-acuity pediatric ED patients. In our urban, academic ED, for example, providers’ rates of diagnostic testing vary by approximately 10-fold. Physician practice variability contributes to prolonged treatment times during low-acuity ED visits.^1^ Patient satisfaction and overall ED throughput are strongly related to timeliness of care.^2,3^ In addition to delivering high-quality care, providers are particularly responsible for efficient care for low-acuity patients. Hospital admission, specialty consultation, and extensive diagnostic workups are rarely necessary.^1^ Effort to reduce diagnostic testing for low-acuity visits in the pediatric ED can be successful without worsening patient outcomes.^4^

A reduction in diagnostic testing for low-acuity patients would likely be safe and desirable. However, decreasing practice variability is one of the more challenging components of streamlining care delivery in the ED. Techniques such as computerized clinical practice guidelines or standardized clinical assessment and management plans are subject to human, local environmental, and technological factors that can affect compliance.^5^ At our center, provider variability specific to diagnostic testing remains high despite efforts to educate low-acuity providers and offer feedback on their performance compared to local benchmarking. Computer modeling can demonstrate the impact of provider change and illustrate the importance of these behavioral changes on ED leadership and individual providers.

### Importance

Computer simulation modeling for healthcare systems and processes shows promise for attending to healthcare problems, such as patient flow and resource utilization.^6^ Discrete event simulation (DES) modeling represents the behavior of a complex, real-world system within a mathematical model that produces quantitative reports to better understand the performance of the real-world system.^7,8^ DES was used in the ED setting as early as the 1980s to compare different nursing schedules and predict the wait times for patients based on each schedule.^9^ More recent work with DES has evaluated the impact that adding a provider to ED triage can have on the length of stay (LOS).^10^

DES is particularly well-suited for improvement efforts that rely on ED leadership to support individual providers in changing their behavior. Quantitative outputs from a DES model can replace speculation to promote investment by stakeholders at every level during a quality improvement effort.^11^ Model estimates can help ED leadership evaluate whether the clinical impact of decreasing variability in diagnostic testing is worth investing additional time and energy. Individualized reports, including provider-specific model outputs, represent a novel opportunity to motivate behavior change and reduce diagnostic testing.

### Study Goal

Our primary goal is to illustrate the application of DES methodology to a real-world QI initiative. To demonstrate the application of DES, we chose a quality improvement project to decrease the LOS of low-acuity patients presenting to the ED by reducing variability in provider diagnostic testing rates.

### Modeling Objective

We sought to leverage the advantages of DES to test the impact of theoretical reductions in diagnostic testing rates on mean LOS in a pediatric ED.

## METHODS

### Study Setting and Population

The setting was a large, urban, tertiary, academic pediatric ED, and level 1 pediatric trauma center with approximately 90,000 annual visits. The pediatric ED is a two-track system with a high-acuity area staffed by fellowship-trained pediatric emergency medicine faculty, fellows, and resident trainees. The study population was low-acuity patients, defined as Emergency Severity Index (ESI) triage level 4 or 5. These patients are predominantly treated in a separate area staffed by pediatricians, nurse practitioners, physician assistants, and occasionally pediatric emergency medicine fellows and attendings working extra shifts for supplemental income. This project was undertaken as a quality improvement initiative; it did not constitute human subjects research. Therefore, it did not require review and approval from the institutional review board review.

### Data Collection

**Fig. 1.**
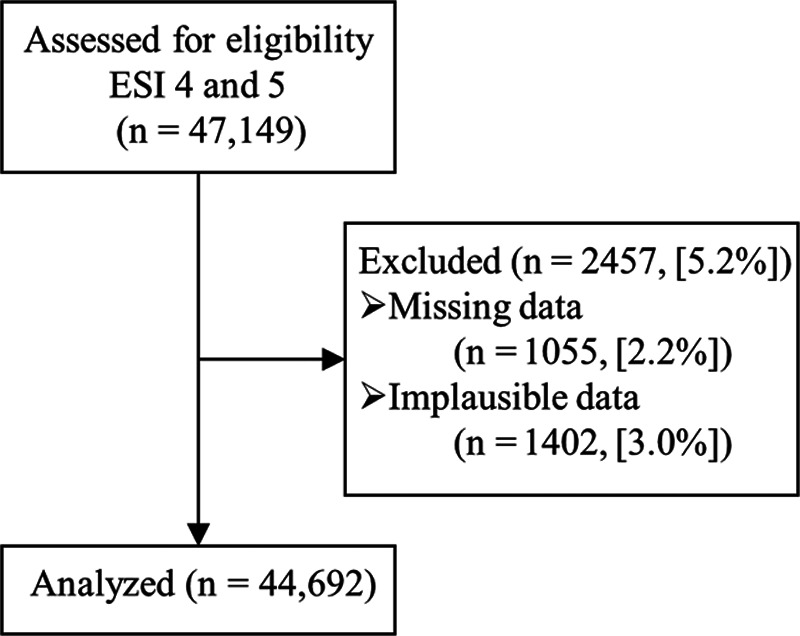
Low-acuity visits from July 1, 2017, to June 30, 2018 were included in the modified model’s development.

We extracted all data from the electronic medical record (EMR) and the ED tracking system (Cerner FirstNet, Cerner Corporation, Kansas City, Mo.). Data were obtained retrospectively for low-acuity patients arriving between July 1, 2017, and June 30, 2018. Collected data included the number of patients, arrival time, time seen by a provider, diagnostic testing, and disposition time. Patients missing any of these data points in the EMR were excluded from this analysis (Fig. 1). Consistent with our usual practice for data cleaning, we excluded patients with implausible data caused by computer entry errors.

### Main Measures and Outcomes

**Fig. 2.**
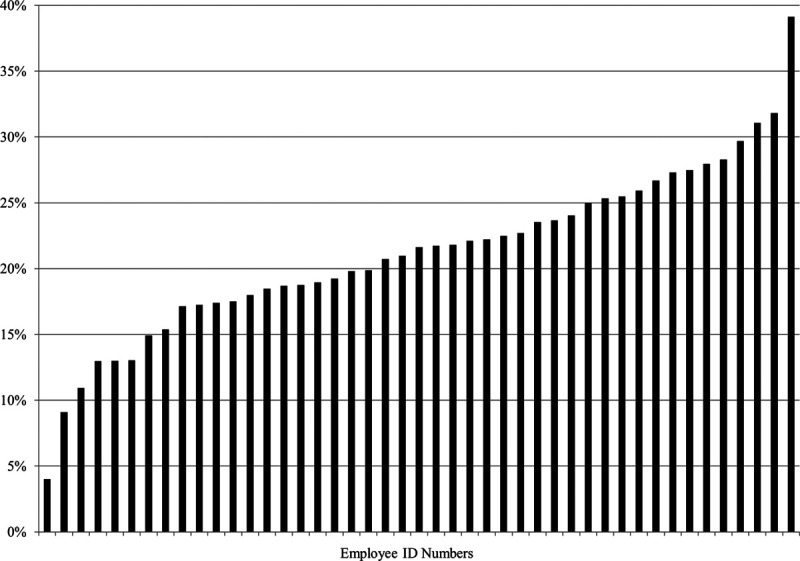
Biannual provider feedback: Percentage of patient visits with diagnostic testing by provider. July 1–December 31, 2019.

This modeling project is part of a broader initiative at our institution to improve patient throughput by decreasing unnecessary radiology and laboratory testing, which includes the provision of guidelines for testing, local benchmarking, audits, and feedback (Fig. 2).

Our key modeling outcome measure was LOS reduction for ESI 4 and ESI 5 patients. In conversation with low-acuity providers and our low-acuity Fast Track director, we set an a priori decrease in LOS of 15 minutes, roughly 10% of current mean LOS, as clinically impactful. We defined LOS for each patient as the time from arrival to disposition. The secondary measure was the wait time, which we defined as the time from arrival until the beginning of evaluation by a licensed independent practitioner.

### System Description and Conceptual Modeling

**Fig. 3.**
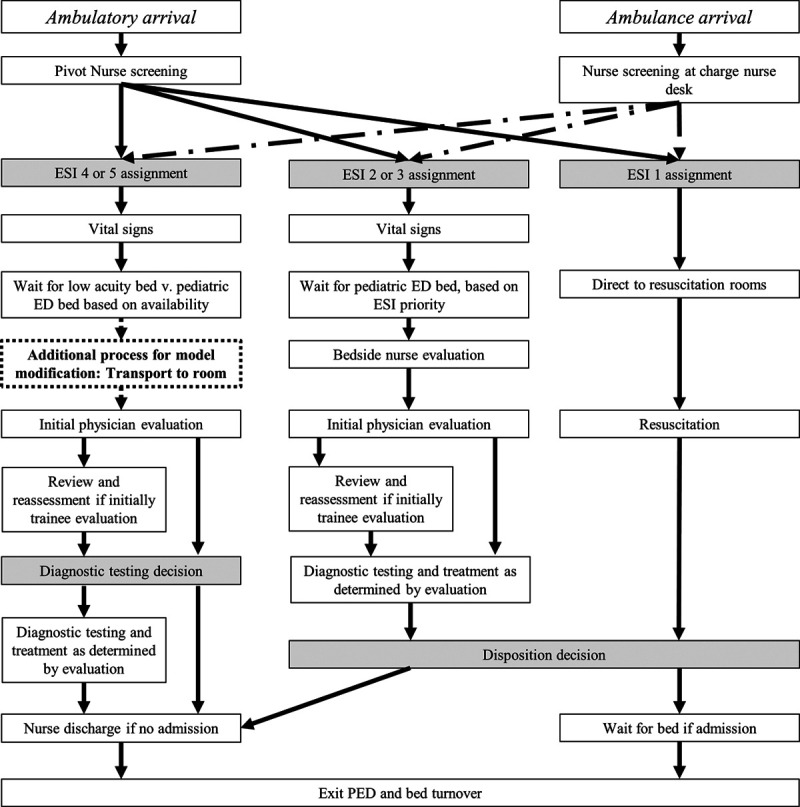
Conceptual model of patient flow.

We modified an existing model built for another large, urban, academic pediatric ED. We updated local testing rates for ESI 4 and 5 patients and added additional processes suggested by local experts (Fig. 3).

### Parameters for Prior Model

The prior model was developed and validated at Morgan Stanley Children’s Hospital (MSCH) of New York Presbyterian.^12^ Resources in the model, including available ED rooms and staff, were based upon direct experience and staff schedules at MSCH and confirmed by conversations with physician and nursing leadership. Probability distributions, used to represent evaluation and treatment times in the model mathematically, were derived from prospective observations and retrospective data at MSCH and previously published process durations. The proportion of patients undergoing testing in the model was set as a fixed proportion for each ESI, and patients without testing were discharged after evaluation. The prior model was validated against actual clinical data at MSCH for mean LOS and wait times and for each ESI 1-5.

### Parameters for Modified Model

We used mean testing rates by providers at our center, collected from administrative data between July 1, 2017, and June 30, 2018, to modify the proportion of patients undergoing testing in the model. We surveyed a group of low-acuity providers to generate notional values to define a probability distribution that could be used to describe the transfer-to-room process’s duration mathematically. This process was identified as the most significant difference between the prior model and our system.

### DES Modeling and Validation of the Modified Model

We used Arena 16.0 (Rockwell Automation, Coraopolis, Pa.), the same software environment used for the prior model, to modify the DES model for this project. The model represents a patient’s journey through a pediatric ED as a series of processes with assigned durations defined using probability distributions. Overall patient volumes, acuity distributions, and pediatric ED resource allocation was representative of the pediatric ED on which the prior model was based. These parameters were not altered to modify the model. Still, the proportion of patients receiving testing was updated to reflect our center, and a transfer-to-room process was added to the modified model. We then verified the model by observing a computer representation of simulated patients in the modified model to confirm they flowed from process to process as expected in our pediatric ED. Validation was performed by comparing model output predictions of mean LOS and wait times to actual site-specific data for ESI 4 and 5 patients for 1 year.

### Model Experiments and Outcome Measures

For all modeling, we simulated 5 iterations of 1 year of patients flowing through our system. Our primary analysis compared mean LOS and wait times between a model with current testing rates to a model with testing based on achievable benchmark testing rates. We calculated an achievable benchmark of care using Achievable Benchmark of Care (ABC) methodology^13^ for ESI 4 and ESI 5 patients, respectively (**Supplemental Digital Content 1**, which describes achievable benchmark of care calculation, http://links.lww.com/PQ9/A249). This methodology measures the actual performance achievable within a given system by examining the top performers. The researchers who developed the ABC methodology have established the effectiveness of achievable benchmarks for delivering provider feedback to improve performance.^14^

We recorded the mean LOS and wait times for ESI 4 and 5 patients from each iteration and compared the models using descriptive statistics. We also generated throughput predictions from a model with testing set at high levels, defined as the diagnostic testing rate of the 90th percentile provider. We tested additional scenarios with diagnostic testing rates set at the highest-testing provider level and diagnostic testing rates at 100% for ESI 4 and ESI 5 patients.

## RESULTS

### Model Parameters

The transfer-to-room was the only missing process identified. The probability distribution parameters used for this process are reported in Table 1, along with other model parameters based upon retrospective data and direct observations in another large, urban, academic pediatric ED. We updated testing rates to represent current local rates (22.5% of ESI 4 and 11.9% of ESI 5 patients) between July 1, 2017, and June 30, 2018.

**Table 1.** Theoretical Probability Distributions of Pediatric ED Process Durations

Pediatric ED Process	Data Source*	Service Time *Distribution* (Parameters), min
Parameters from prior model		
Patient arrival	Retrospective	*Poisson* (λ†)
Nurse screening	Observations	*Gamma* (0.567, 4.9)
Vital signs	Observations	1 + *Gamma* (1.58, 1.51)
Attending physician evaluation	Retrospective	2 + 28 × *Beta* (0.726, 1.08)
Resident physician evaluation	Retrospective	2 + 28 × *Beta* (1.11, 1.04)
Attending/resident review	Literature	−0.001 + *Weibull* (6.4, 1.27)
Attending physician reassessment	Literature	−0.001 + *Exponential* (3.88)
Testing and treatment ESI 4 patients	Retrospective	5 + *Gamma* (136, 0.874)
Testing and treatment ESI 5 patients	Retrospective	5 + *Weibull* (45.1, 0.701)
Nurse discharge process	Expert Opinion	*Triangular* (5, 10, 15)
Bed cleaning	Literature	2 + *Lognormal* (17.4, 16.2)
Updated parameters for modified model		
Transfer to room (ESI 4, 5)	Expert Opinion	*Triangular* (5, 10, 12)
Proportion patients undergoing testing	Retrospective	Fixed proportion for each ESI

*Theoretical probability distributions were based on the best available data for each process: Retrospective = retrospective review of single-visit level data from another large, urban, academic pediatric ED for development of prior model; data obtained from 36 randomly selected dates in 2016, including processes for which clear start and stop times were included in the electronic medical record. Observations = direct observations, at another large, urban, academic pediatric ED for development of prior model; centered on patient triage processes for which limited electronic medical record data were available. Literature = previously published distributions based on direct observations of pediatric ED processes. Expert Opinion = local expert estimates of the duration of a process for which no retrospective or previously published data were available.

†Variable arrival rate with distinct λ each hour, ranging from 1.63 to 8.56 patients per hour.

### Model Verification and Validation

After the addition of the transfer-to-room process, a team of local experts verified that patients flowed through the model from process to process in the order that would be expected in the real-life system. Validation against actual clinical data for 1 year demonstrated good model fit, with no statistically significant differences in mean LOS or wait times (Table 2) for ESI 4 and ESI 5 patients.

**Table 2.** Validation of Outcome Measures for Modified Model

	Model with Current Testing	Administrative Data (FY 2018)	Difference between Model Output and Administrative Data
ESI	Mean LOS in min (SD)	Mean LOS in min (SD)	Difference in LOS in min (95% CI)
4	156.8 (6.2)	169.8 (21.4)	−13.0 (−34.1, 8.1)
5	133.3 (5.2)	136.4 (27.8)	−3.1 (−30.3, 24.1)
ESI	Mean WT in min (SD)	Mean WT in min (SD)	Difference in WT in min (95% CI)
4	87.6 (7.0)	76.4 (22.0)	11.2 (−10.6, 33.0)
5	85.1 (6.3)	73.6 (25.1)	11.5 (−13.2, 36.2)

FY, fiscal year.

### Model Experimentation Outcomes

Compared to a model with current testing, the mean LOS with low benchmark testing was significantly shorter for both ESI 4 (difference 19.1 min [95% confidence interval 12.2, 26.0]) and ESI 5 (10.9 min [4.5, 17.3]) patients (Table 3). However, the point estimate for reduced LOS for ESI 5 patients was less than our team determined, a priori to be clinically meaningful. Using point estimates from the models with benchmark rates compared to current testing rates, 35,838 ESI 4 patients would be present in the ED for 11,468 fewer hours. The 9,124 ESI 5 patients who presented over 1 year would spend a total of 1,658 fewer hours in the pediatric ED in a scenario with benchmark testing. In models with diagnostic testing set at higher rates, LOS for low-acuity patients increased with increased testing rates.

**Table 3.** Outcome Measures Summary For Models with Current and ABC Testing

	Model with Current Testing*	Model with ABC Testing	
ESI	Mean LOS min (SD)	Mean LOS min (SD)	Difference in LOS min (95% CI)
4	156.8 (6.2)	137.7 (2.4)	19.1 (12.2, 26.0)
5	133.3 (5.2)	122.4 (3.4)	10.9 (4.5, 17.3)
ESI	Mean WT min (SD)	Mean WT min (SD)	Difference in WT min (95% CI)
4	87.6 (7.0)	78.7 (2.0)	8.9 (1.4, 16.4)
5	85.1 (6.3)	77.7 (2.30)	7.4 (0.5, 14.3)

*Current testing based on local rates (22.5% of ESI 4 and 11.9% of ESI 5 patients) between July 1, 2017, and June 30, 2018. Achievable benchmark calculated during the same time period as testing 13.5% and 4.2% of ESI 4 and ESI 5, respectively.

## DISCUSSION

In this study in a large, urban, academic hospital, DES modeling predicted a statistically significant and clinically impactful decrease in mean LOS for ESI 4 patients if diagnostic testing is performed at achievable benchmark rates compared to current rates. The model also predicts a statistically significant but lower clinical impact improvement for ESI 5 patients. Achieving benchmark diagnostic testing could decrease patients’ total time in the ED each year by many thousands of hours.

ED crowding, the most severe problem related to ED patient throughput, is widely recognized as a secondary result of inpatient crowding, which cannot always be impacted by stakeholders in the ED.^15,16^ However, diagnostic testing in the ED, including laboratory and radiology testing, is within ED providers’ control and is associated with longer ED LOS and wait times.^17–20^ To our knowledge, no prior studies use complex computational modeling to estimate the impact of decreasing diagnostic testing rates on low-acuity ED patient throughput. Our modeling approach to this problem is supported by decades of use of DES to address ED staffing and scheduling decisions in the healthcare setting.^9,10,21^

Modeling proposed system changes can help estimate the magnitude of beneficial impacts before undertaking efforts to effect changes. In the context of healthcare improvement, DES models can generate predictive diagnostic data and compare the impact of different improvement options in a theoretical system before implementation.^11^ In addition to applying our findings to understand the potential benefit of a reduction in diagnostic testing, we can use our DES model to compare scenarios with individual providers to estimate the impact of their diagnostic testing decisions on system performance. In this way, we can use our model to encourage individual providers to minimize unnecessary testing and motivate our entire provider group to be more deliberate in ordering tests.

## LIMITATIONS

This study has several limitations. First, we modified an existing model built to represent a large, urban, academic, pediatric ED with patient flow patterns that we determined to be similar to our ED. We did not independently confirm the service times for all processes in the model. Therefore, the model does not represent the system we are trying to study in granular detail but does represent the primary function of a large, urban, academic pediatric ED.

Validation of model outputs for mean LOS and wait times against actual clinical data support this model’s use to study patient throughput in our setting. However, we did not evaluate how accurately our model predicts decreased LOS for a given reduction in diagnostic testing in the real-world, nor did we establish the efficacy of our planned QI intervention. Further study is needed to confirm the real-world reproducibility of our model predictions.

Decisions made during model development can affect the accuracy of model predictions. For example, we did not model the dynamic effects of specific essential resources that might change with improved diagnostic testing rates. Some resources, such as the availability of a radiology technician or a lab technician, would likely become less of a constraint on patient flow as decreasing proportions of patients are assigned these tests. The overall effect of this example is that the model predictions likely underestimate the real impact of testing. Our model, incorporating patient competition for bed, nursing, and provider resources, still has significant advantages over any estimate that ignores all ED resource constraints (**Supplemental Digital Content 2**, which describes arithmetic, http://links.lww.com/PQ9/A249).

Finally, we do not have enough data to predict how a reduction in diagnostic testing might impact the rate of missed diagnoses or the number of return visits to the ED. At our institution, providers are already notified of each patient they see that returns within seven days of ED discharge. As part of any effort to decrease LOS by changing provider behavior, we will carefully follow the return visits rate as a balancing measure. We will also seek regular provider input to explore concerns about missed diagnoses and other perceived barriers. Emails with individualized performance reports will include a request for feedback regarding personal concerns or anticipated challenges that might prevent a reduction in testing to our achievable benchmark.

## FUTURE STEPS

As local stakeholders decrease LOS by reducing provider variability in diagnostic testing, we will closely track changes to mean LOS and diagnostic testing rates. We plan a future report on our model predictions’ real-world reproducibility, setting testing in the model equal to the overall diagnostic testing rate we achieve through improvement efforts.

We also plan to use the model as a tool to motivate provider behavioral change. Provider behavioral change techniques are more likely to be successful if based in theory and supported by evidence.^22^ Our modeling approach provides evidence for the benefits for patients of decreased diagnostic testing. We plan to provide individualized reports (**Figure 1, Supplemental Digital Content 3**, http://links.lww.com/PQ9/A248) to help providers conceptualize the costs of elevated testing rates in terms of length of stay and crowding. Both extrinsic and intrinsic motivators are essential for changing provider behavior.^23^ Individualized feedback to providers on the impact they can have on a patient-centered outcome, such as LOS, could contribute to intrinsic motivators to effect individual behavioral change and ultimately achieve lower testing rates across all providers.

## CONCLUSIONS

A modified DES model of a large, urban, academic pediatric ED provided insight into the expected system benefit of decreasing diagnostic testing rates for low-acuity patients to an achievable benchmark level. Model output predictions support the investment of resources to work toward this benchmark as a means of decreasing LOS for ESI 4, and to a lesser extent, ESI 5 patients. The same model can generate provider-specific reports to quantify that provider’s impact and build intrinsic motivation for provider behavior change. Future study is needed to evaluate the real-world reproducibility of model predictions and estimate the cost-saving and resource-sparing benefits associated with a reduction in diagnostic testing, which might be especially important during a crisis.

## DISCLOSURE

The authors have no financial interest to declare in relation to the content of this article.

## ACKNOWLEDGMENTS

The authors would like to acknowledge Dr. Cindy G. Roskind, Dr. John Babineau, and Dr. F. Meridith Sonnett, who were critical in building the prior model used for this study.

## Supplementary Material


