# Supplementary figures and images for: Reducing Pediatric ED Length of Stay by Reducing Diagnostic Testing: A Discrete Event Simulation Model
Source: Pediatr Qual Saf. 2021 Mar 10;6(2):e396. doi: 10.1097/pq9.0000000000000396 (PMC7952107; doi:10.1097/pq9.0000000000000396)

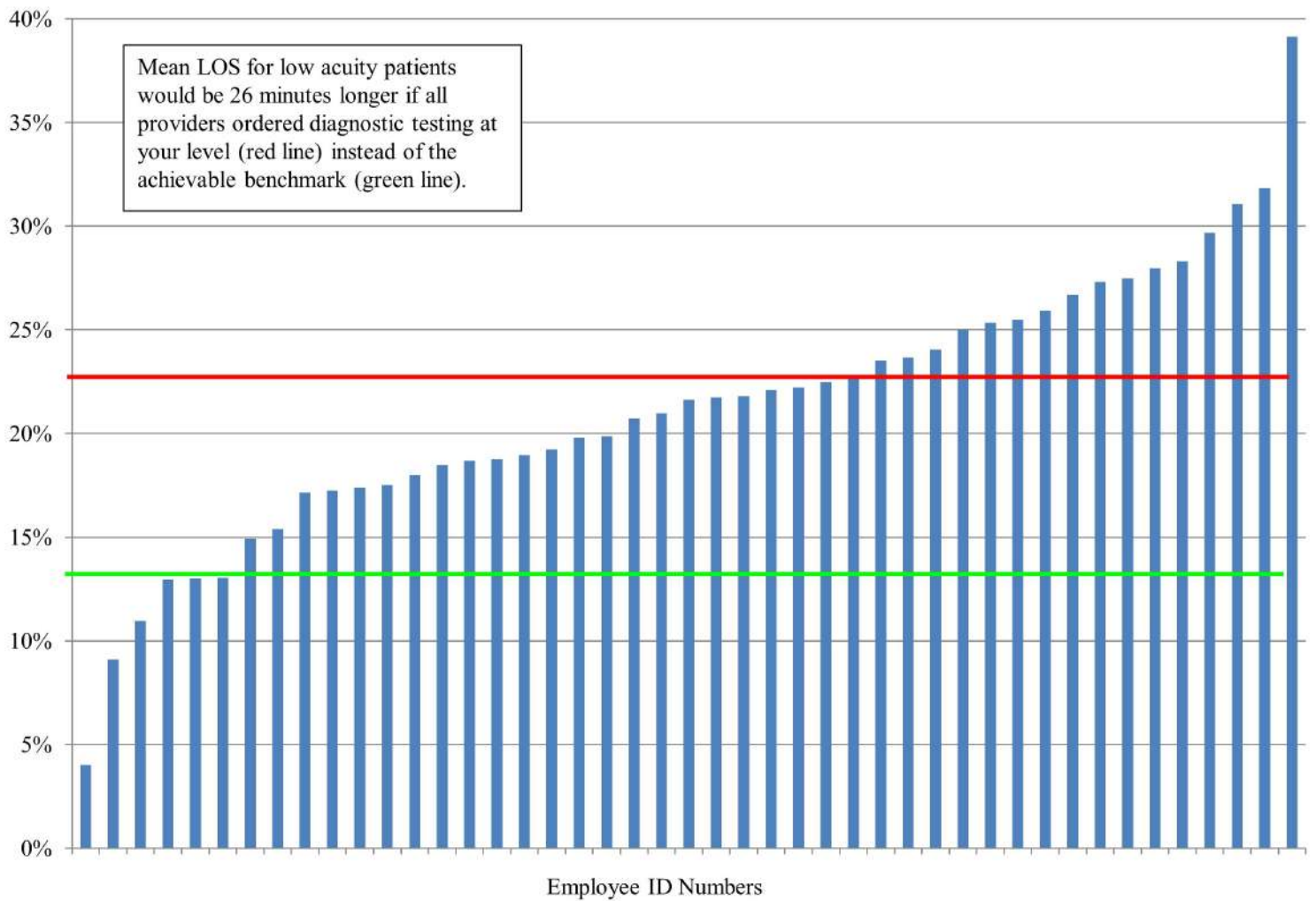

**SDC Figure 1.** Proposed Provider Report  
LOS = Length of Stay

Supplement: Supplementary file 1 [file pqs-6-e396-s001.pdf]
