# Supplementary material for: Reducing Pediatric ED Length of Stay by Reducing Diagnostic Testing: A Discrete Event Simulation Model
Source: Pediatr Qual Saf. 2021 Mar 10;6(2):e396. doi: 10.1097/pq9.0000000000000396 (PMC7952107; doi:10.1097/pq9.0000000000000396)
Supplement: Supplementary file 2 [file pqs-6-e396-s002.pdf]

Title: Reducing Pediatric ED Length of Stay by Reducing Diagnostic Testing: A Discrete Event Simulation Model

McKinley KW, et al.

SUPPLEMENTARY DIGITAL CONTENT

Achievable Benchmark of Care Calculation

We calculated an Adjusted Performance Fraction (APF), using a Bayesian correction to reduce the impact of providers with small numbers of eligible patients[a,b]

APF = (x + 1)/(d + 2),

where x is the actual number of low acuity patients receiving no diagnostic workup, and d is the total number of low acuity patients.

1. Based on the Bayesian Adjusted Performance Fraction (APR), we ranked providers, including PAs and physicians, in descending order of performance for this specific quality indicator
2. We sequentially added providers in descending order, starting with the best-performing provider, until we had a subset of providers representing at least 10% of all patients in the entire dataset
3. Retaining the APF ranking, we based Step 4 on crude data
4. We calculated the achievable benchmark of care:

(Total number of patients in subset receiving no diagnostic testing) / (Total number of patients in subset)

We performed these steps separately for Emergency Severity Index (ESI) 4 and 5 patients.

Sensitivity Analysis

METHODS – Since the transfer-to-room process was based on local expert opinion without direct observations, we performed sensitivity analyses to assess how varying the duration of this process would impact our results. We repeated out our analyses with the transfer-to-room process set at zero (as in our prior model) and with the transfer-to-room process set to 150% of the notional estimate.

RESULTS – Increasing or decreasing the duration of the transfer-to-room process caused an increase and decrease, respectively, in the mean LOS of low acuity patients. The relative impact of current versus benchmark testing was similar to our primary analysis (**SDC, Table 1**). In our first sensitivity analysis with negligible transfer-to-room durations, the mean LOS with benchmark testing was shorter for both ESI 4 (difference 11.6 mins [95% CI 7.9, 15.3]) and ESI 5 (5.5 mins [1.2, 9.8]) patients compared to a model with mean testing. In our second sensitivity analysis with increased transfer-to-room durations, the mean LOS with benchmark testing remained shorter for ESI 4 (13.4 mins [95% CI 5.9, 20.9]) and ESI 5 (8.0 mins [1.1, 14.9]) patients compared to a model with increased transfer-to-room durations and mean testing. The results of these sensitivity analyses suggest that the models are robust to changes in assumptions about the transfer-to-room process.

Table 1. Sensitivity analyses with varying duration of transfer-to-room process. Outcome measures summary for models with and current and ABC^{TM} testing.

	Model with current testing, negligible transfer-to-room	Model with ABC^{TM} testing, negligible transfer-to-room	
ESI	Mean LOS min (SD)	Mean LOS min (SD)	Difference in LOS min (95% CI)
4	106.8 (4.4)	93.4 (5.8)	13.4 (5.9, 20.9)
5	81.9 (4.8)	73.9 (4.6)	8.0 (1.1, 14.9)
	Model with mean testing, transfer-to-room increased by 150%	Model with ABC^{TM} testing, transfer-to-room increased by 150%	
ESI	Mean LOS min (SD)	Mean LOS min (SD)	Difference in LOS min (95% CI)
4	218.1 (2.7)	206.5 (2.3)	11.6 (7.9, 15.3)
5	195.1 (2.1)	189.6 (3.6)	5.5 (1.2, 9.8)

ABC = Achievable Benchmark of Care

LOS = Length of Stay

Arithmetic Approximation

Using an arithmetic approximation, we calculated the minimum expected improvement to throughput if all providers performed at benchmark levels for diagnostic testing. For this approximation, we assumed no competition for ED resources. We further assumed that a change in the proportion of patients receiving testing would not alter the mean length of stay (LOS) for patients receiving diagnostic testing or the mean LOS for patients discharged with no testing.

We multiplied the mean LOS for ESI 4 patients receiving diagnostic testing at our site by our achievably low testing benchmark. We then multiplied the mean LOS for ESI 4 patients discharged without testing by [1 – achievable benchmark]. We added the products of these calculations to approximate LOS for ESI 4 patients. We repeated this arithmetic for ESI 5 patients and then performed the same calculations with the proportions for diagnostic testing set at current rates.

Between July 1, 2017 and June 30, 2018, the mean LOS for patients at our center that had diagnostic testing done was 218.9 (SD 90.7) and 200.7 (SD 85.5) minutes for ESI 4 and ESI 5, respectively. Patients without testing done had a LOS 158.1 (SD 75.5) minutes for ESI 4 and 133.7 (SD 67.4) for ESI 5. Based on our arithmetic approximation, described above, there would be a decrease in mean LOS of 5.5 (ESI 4) and 5.2 (ESI 5) minutes if all providers tested at the achievably low benchmark rate rather than current rates. Because our arithmetic approximation assumes no competition for ED resources, it does not provide a complete picture of the system cost of diagnostic testing for low acuity patients.

Data Supplement References

a. Agresti A. *Categorical Data Analysis*. New York, NY: John Wiley & Sons; 1990.

b. Kiefe CI, Weissman NW, Allison JJ, Farmer R, Weaver M, Williams OD. Methodology matters-XII. Identifying achievable benchmarks of care: Concepts and methodology. *Int J Qual Health Care*. 1998;10(5):443-447. doi:10.1093/intqhc/10.5.443
